# p62-Induced Cancer-Associated Fibroblast Activation via the Nrf2-ATF6 Pathway Promotes Lung Tumorigenesis

**DOI:** 10.3390/cancers13040864

**Published:** 2021-02-18

**Authors:** Ji In Kang, Dong Hyun Kim, Ki Woon Sung, Sang Mi Shim, Hyunjoo Cha-Molstad, Nak Kyun Soung, Kyung Ho Lee, Joonsung Hwang, Hee Gu Lee, Yong Tae Kwon, Bo Yeon Kim

**Affiliations:** 1 Anticancer Agents Research Center, Korea Research Institute of Bioscience and Biotechnology, Ochang, Cheongwon 28116, Korea; jik6848@naver.com (J.I.K.); waikiki@kribb.re.kr (D.H.K.); hcha@kribb.re.kr (H.C.-M.); soungnak@kribb.re.kr (N.K.S.); leekh@kribb.re.kr (K.H.L.); hwangj1@kribb.re.kr (J.H.); 2 Department of Biomolecular Science, University of Science and Technology, Daejeon 34113, Korea; 3 Protein Metabolism Medical Research Center, Department of Biomedical Sciences, College of Medicine, Seoul National University, Seoul 03080, Korea; kwsung02@snu.ac.kr (K.W.S.); absti32@snu.ac.kr (S.M.S.); 4 CuePeakbio Co., Ltd., Rm 310, Venture Company Bd, Korea Research Institute of Bioscience & Biotechnology, Daejeon 34141, Korea; 5 Immunotherapy Research Center, Korea Research Institute of Bioscience and Biotechnology, Daejeon 34141, Korea

**Keywords:** tumor microenvironment, cancer-associated fibroblast, lung adenocarcinoma, p62/SQSTM1/Sequestosome-1, selective autophagy, nuclear factor erythroid 2-related factor 2, activating transcription factor 6

## Abstract

**Simple Summary:**

Cancer-associated fibroblasts (CAF) arise from normal fibroblasts within the tumor microenvironment (TME) and promote tumorigenesis through metabolic reprograming and secretion of tumor promoting molecules such as transforming growth factor beta (TGFβ). Here, we show that autophagy plays a key role in CAF activation. During CAF activation, fibroblasts induce the mRNA expression of *p62*, and resulting p62 targets Keap1 for lysosomal degradation, which allows the nuclear translocation of Nrf2 and transcriptional induction of antioxidant responses. The transcriptional targets of Nrf2 include ATF6, which mediates ER stress responses. Taken together, normal fibroblasts are differentiated into CAFs as protective responses to stresses under TME via the p62-Nrf2 pathway.

**Abstract:**

Cancer-associated fibroblasts (CAFs) are important in tumor progression. The autophagy adaptor protein, p62/SQSTM1/Sequestosome-1, is up-regulated in tumors, but down-regulated in CAFs in the early stages of lung adenocarcinoma. We investigated whether p62-induced autophagy might control CAF activation. Under CAF-inducing conditions, like hypoxia or cancer cell co-cultures, p62 ablation or autophagy inhibition with hydroxychloroquine (HCQ) impaired CAF activation and reduced transforming growth factor beta (TGFβ) production, which impeded tumor growth. During CAF activation, p62-induced autophagy up-regulated the expression of the anti-oxidant signaling protein, nuclear factor erythroid 2-related factor 2 (Nrf2), and the ER-stress response regulator, activating transcription factor 6 (ATF6). Genetically or pharmacologically inhibiting the Nrf2-ATF6 pathway totally blocked CAF activation and tumor progression. These results demonstrate that p62 is a key modulator of primary lung adenocarcinoma progression. Thus, targeting the p62-Nrf2 autophagy signaling pathway might be a novel, stroma-focused, cancer prevention and/or treatment strategy.

## 1. Introduction

Lung cancer is the most common cancer type and the leading cause of cancer-associated death world-wide [1]. Nearly 80% of lung cancers are classified as non-small cell lung cancers, including adenocarcinoma [2]. Traditional anti-cancer strategies have targeted cancer cells separately; however, tumorigenesis occurs in a complex microenvironment called the tumor microenvironment (TME). The TME is crucial for the development of primary tumor progression, drug resistance, and metastasis [3].

Cancer-associated fibroblasts (CAFs) are the most abundant cell type in the TME [3]. CAFs predominantly arise from normal fibroblasts under the influence of tumor derived stimuli, such as low nutrients, hypoxia, reactive oxygen species (ROS), and inflammatory signals [4,5]. CAFs play essential roles in supporting primary tumor growth, angiogenesis, metastasis, and therapy resistance [6]. CAFs exhibit a contractile myofibroblast phenotype; they produce matrix proteins and secrete tumor-promoting factors, like growth factors, cytokines, and energy-rich metabolites [3,5]. Tumor progression is driven by transforming growth factor beta (TGFβ), which is essential for the acquisition and maintenance of the CAF phenotype [7]. To date, the detailed molecular mechanisms that underlie the capability of CAFs to promote tumorigenesis remain largely unclear.

The TME is highly similar to autophagy-activating conditions [8]. Autophagy is a homeostatic mechanism responsible for lysosome-dependent degradation of cellular molecules and organelles [9]. In this context, a reverse Warburg effect was proposed, whereby the activation of stromal autophagy by hypoxia activates a glycolytic switch that produces high-energy intermediates, such as lactate [10]. However, this concept remains quite controversial, because the traditional Warburg effect holds true for most types of cancer cells [11]. Recently, another autophagy-related study revealed that the turnover of the transcription factor, CSL/RBPJκ, which depends on the autophagy receptor protein, p62 (SQSTM1/Sequestosome-1), drives CAF activation in skin cancer [12]. However, the association between autophagy and the molecular mechanism involved in the differentiation of healthy fibroblasts into CAFs during tumor progression is not well understood.

The autophagy regulator, p62, is a selective cargo receptor that facilitates the degradation of specific proteins, including the Kelch-like ECH-associated protein 1 (KEAP1) and the heat shock protein family A member 5 (HSPA5/GRP78/BiP). The degradation of KEAP1 activate anti-oxidant signaling through nuclear factor erythroid 2-related factor 2 (Nrf2). The degradation of HSPA5 activated the cellular stress response [13,14]. p62 is constitutively degraded by autophagy, and autophagy inhibition leads to an increase in p62 aggregates [15]. The loss of p62 in CAFs has been reported in several types of cancer, such as prostate cancer and liver cancer [16,17]. The loss of p62 in prostate cancer CAFs has been implicated in metabolic reprogramming through an mTORC1/Myc pathway that regulates the production of transforming growth factor beta (TGFβ) [16]. In the liver, the loss of p62 activates stromal stellate cells by impairing vitamin D receptor (VDR) signaling [17].

Nrf2 is a critical transcription factor that regulates the cellular antioxidant response by activating genes that contain an antioxidant response element (ARE, 5′-GTGACNNNGC-3′) [18]. The Nrf2 signaling pathway is negatively regulated by the E3 ligase, Keap1, which stimulates the ubiquitination and proteasomal degradation of Nrf2 [19]. Under basal conditions, Nrf2 levels remain relatively low. However, under oxidative stress conditions, cysteine residues in Keap1 are oxidized through the canonical mechanism, which results in a reduction in Nrf2 ubiquitination and an increase its nuclear translocation and activation [20]. Another mode of Nrf2 regulation involves phosphorylated p62 (S349), which can disrupt the Nrf2-Keap1 complex by directly interacting with Keap1 and inducing the autophagic degradation of Keap1; this results in the prolonged activation of Nrf2 [13,21]. In skin fibroblasts, Nrf2 activation induces cellular senescence and the expression of genes characteristic of CAFs [22]. Nrf2 has been considered a promising candidate for cancer treatments, but little is known about the control of the Nrf2 signaling pathway in the TME, particularly its regulation by p62.

Here, we address the role of p62 during CAF activation in lung adenocarcinoma. We found that, during CAF activation, p62 was down-regulated. We also found that, upon p62-induced selective autophagy, Nrf2 was up-modulated, which increased the expression of anti-oxidant genes. Unexpectedly, we found that this p62-Nrf2 pathway could mediate ATF6 activation, which increased the expression of ER-stress response genes. All these sequential signaling pathways were required for CAF activation and the production of the growth factor, TGFβ, which promoted tumorigenesis.

## 2. Results

### 2.1. High p62 Expression Level in CAF-Rich Tumors

**Figure 1:**
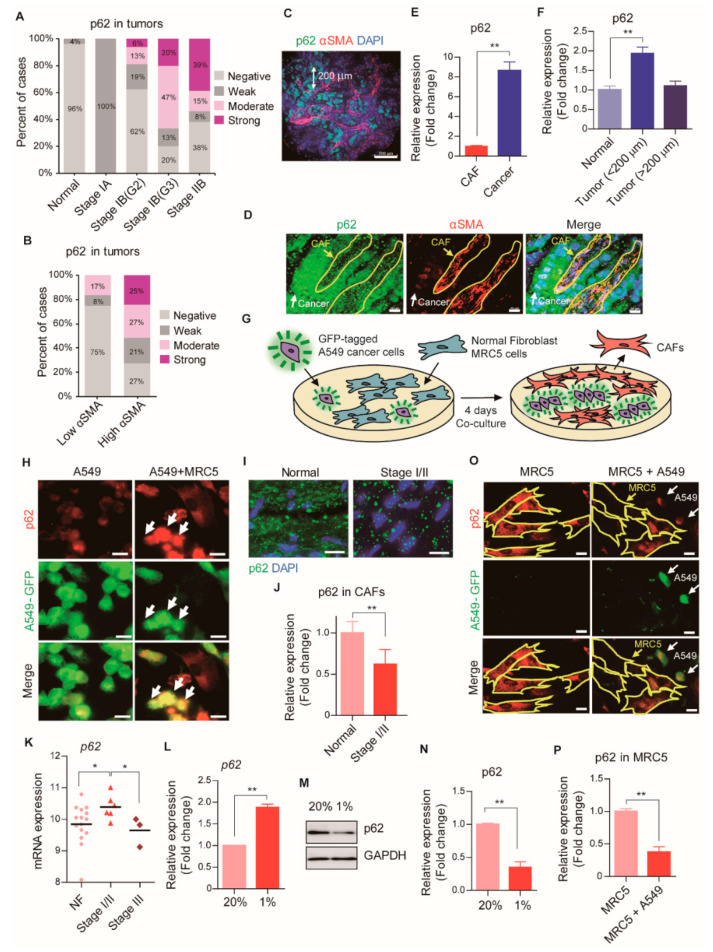
Post-translational loss of p62 during CAF activation. (**A**–**F**) Immunofluorescence analysis of p62 expressions in the tumors of 46 stage I/II lung adenocarcinoma patients using a tissue array. αSMA-stained CAFs were distinguished from unstained cancer cells or normal cells. (**A**) The association between p62 expression levels (negative, weak, moderate, and strong) and cancer stage. G2 and G3: grading states. (**B**) The correlation between p62 and αSMA expressions. (**C**,**D**) Representative images showing strong p62 expressions in CAF-adjacent tumors. CAFs were surrounded with yellow line and cancer cells were indicated with white arrows. Scale bars: (**C**) 200 μm, (**D**) 20 μm. (**E**) Quantification of intensity signal for p62. Error bars, SD. (**F**) The association between p62 expressions and the distance from CAF (<200 μm or >200 μm). Error bars, SD. (**G**) A schematic representation of CAF differentiation from normal fibroblasts by co-culture with GFP-tagged cancer cells. (**H**) Representative images of immunofluorescence staining revealing p62 expression levels (red) in A549-GFP cells (green, indicated with arrows) after the co-culture with MRC5 cells. Scale bars: 20 μm. (**I**,**J**) Immunofluorescence analysis of p62 expressions in the CAFs of 46 stage I/II lung adenocarcinoma patients using a tissue array. αSMA-stained CAFs were distinguished from unstained cancer cells or normal cells. (**I**) Representative images showing p62 expressions (green) in CAFs. The nuclei were stained with DAPI (blue). Scale bars: 20 μm. (**J**) Quantification of intensity signal for p62. Error bars, SD. (**K**) Analysis of *P62* mRNA expressions in the CAFs of 9 lung adenocarcinoma patients (6 for stage I/II and 3 for stage III). The association between *P62* expression levels and cancer stages. For statistical analysis, two-tailed Mann-Whitney U test was used. Error bars, SD. (**L**) The mRNA expressions of *P62* in MRC5 cells after hypoxia (1% O_2_, 24 h) were analyzed by Real-time RT PCR. GAPDH was used as a reference gene in the analysis. Error bars, SD (*n* = 3). (**M**) The protein expressions of p62 in MRC5 cells after hypoxia (1% O_2_, 24 h) were assessed with Western blot analysis. Full-length blots are presented in Figure S6. (**N**) Quantification of (**M**). Error bars, SD (*n* = 3). (**O**) Representative images of immunofluorescence staining revealing p62 expressions (red) in MRC5 cells (surrounded with yellow line) after the co-culture with A549-GFP cells (green, indicated with white arrows) for 4 days. Scale bars: 20 μm. (**P**) Quantification of intensity signal for p62 in (**O**) Error bars, SD. (* *p* < 0.05, ** *p* < 0.001, *p*-values between depicted groups.).

It has been reported that the accumulation of p62 in patients with lung adenocarcinoma indicated a poor prognosis [23]. Consistently, our immunofluorescence assays with the human lung cancer tissue microarray (46 early stage I/II patient-derived samples) revealed that the levels of p62 protein expression in lung adenocarcinoma were strongly correlated with the stages of initial tumor progression (Figure 1A and Figure S1A). p62 expression was up-regulated in tumors as the severity of cancer progressed. These results suggested that p62 might serve as a potential prognostic marker for the stage of cancer progression.

Previous studies showed that, among patients with lung cancer, those with stroma-rich tumors exhibited worse prognoses than those with stroma-poor tumors [24]. To test whether the number and functions of CAFs were correlated with the expression of p62 in tumors, we co-stained tissue sections with antibodies specific for p62 and the CAF marker alpha smooth muscle actin (αSMA). We found that p62 expression in tumor tissues was specifically marked in the tissues with high αSMA levels (Figure 1B). This result indicated that p62 accumulated in CAF-rich tumors. Interestingly, there was only weak co-localization of p62 and αSMA in CAF-rich tumors, suggesting that p62 expression was increased in cancer cells, not in CAFs (Figure 1C–E). Moreover, tumor cells located within 200 μm of CAFs expressed p62 at higher levels than tumor cells located at a greater distance from CAFs (Figure 1C,F). These results suggested that CAFs might induce p62 expression in cancer cells, which in turn, might promote tumor progression.

To determine the expression of p62 during CAF activation, we co-cultured primary human normal lung fibroblasts (MRC5) with green fluorescent protein (GFP)-labeled human lung cancer cells (A549-GFP) at a 3:1 ratio for four days (Figure 1G). Immunofluorescence analysis showed that co-cultured MRC5 cells induced p62 accumulation in A549 cancer cells (Figure 1H). This result suggested that p62 expression in cancer cells was strongly induced by CAFs. This finding suggested that communication between CAFs and cancer cells could influence the prognosis of patients with cancer, at least in part, through p62.

### 2.2. Post-Translational Regulation of p62 Expression during CAF Activation

Tissue array staining results of lung tumors from patients with early stage I/II cancer also revealed that p62 levels were lower in CAFs than in normal tissues (Figure 1I,J). During the early stages, tumors acquire new blood vessels, which supply nutrients, oxygen, and growth factors; in addition, normal fibroblasts around the tumor differentiate into CAFs, which act as a primary source of nutrients [6]. Therefore, we hypothesized that the loss of p62 in the early stages of cancer might modulate CAF activation, which could contribute to the growth of the primary tumor. To test the correlation between *p62* mRNA levels in CAFs and cancer progression, we analyzed a public mRNA database that comprised 9 CAF samples from patients with lung adenocarcinomas [25]. In contrast to the p62 protein, the *p62* mRNA in CAFs accumulated (*p* < 0.05) during the early stages of cancer (Figure 1K) but, returned to normal levels in the late stages of cancer. We further characterized the role of p62 in the early stages of lung adenocarcinoma by inducing the differentiation of CAFs under hypoxia, a common feature in the TME [26]. Hypoxia induced a marked upregulation of *p62* mRNA, associated with a drastic down-regulation of p62 protein (Figure 1L–N). Consistently, in MRC5 cells co-cultured with A549-GFP cells under normoxic conditions, immunostaining showed a remarkable loss of the p62 protein in CAFs (Figure 1O,P). Similar results were also obtained when the cells co-cultured under hypoxic conditions. These results suggested that p62 was transcriptionally activated and post-translationally down-regulated during CAF differentiation.

### 2.3. p62 Mediates CAF Activation by Enhancing Autophagy

**Figure 2:**
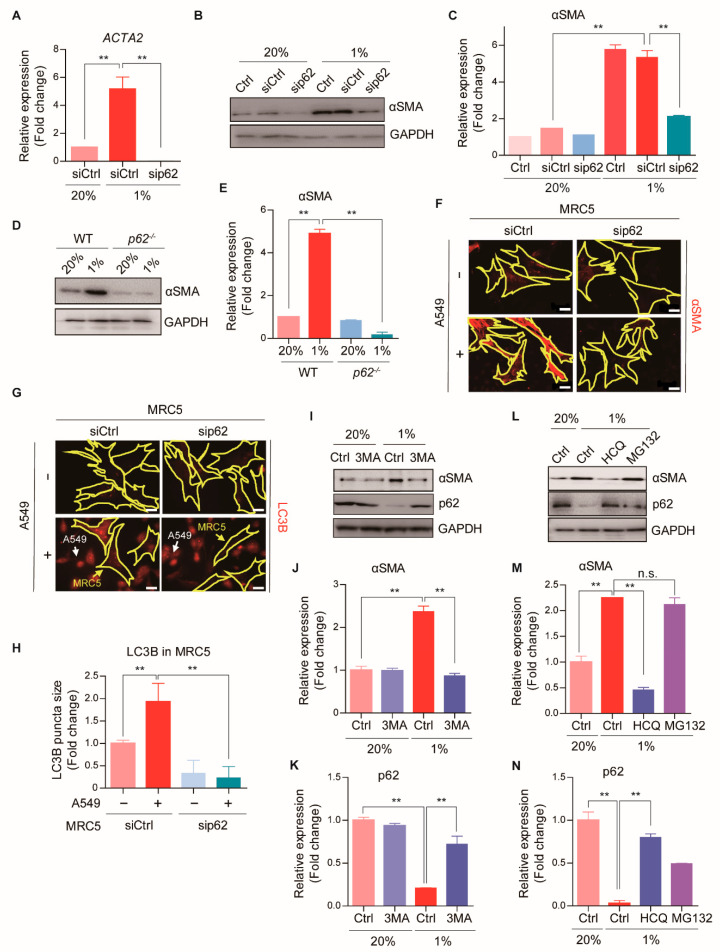
p62-induced autophagy activation during CAF differentiation. (**A**) The mRNA expressions of *ACTA2* after hypoxia (1% O_2_, 24 h) in WT or p62 knock-down MRC5 cells were analyzed by Real-time RT PCR. *GAPDH* was used as a reference gene in the analysis. Error bars, SD (*n* = 3). (**B**–**E**) The protein expressions of αSMA after hypoxia (1% O_2_, 24 h) in WT, (**B**) p62 knock-down MRC5 cells, or (**D**) *p62^−/−^* MEFs were assessed with Western blot analysis. Full-length blots are presented in Figure S6. Quantification of (**C**,**B**) and (**E**,**D**). Error bars, SD (*n* = 3). (**F**–**H**) Immunofluorescence analysis in WT or p62 knock-down MRC5 cells (surrounded with yellow line) after the co-culture with A549-GFP cells (indicated with white arrows) for 4 days. (**F**) Representative images showing αSMA expressions (red). Scale bars: 20 μm. (**G**) Representative images showing LC3B expressions (red). Scale bars: 20 μm. (**H**) Quantification of LC3B puncta size. Error bars, SD (*n* = 3). (**I**–**N**) The protein expressions of αSMA after the treatment of (**I**) 3MA (5 mM, 24 h), (**L**) HCQ (25 μM, 24 h), or MG132 (5 μM, 24 h) in hypoxic (1% O_2_, 24 h) MRC5 cells were analyzed by Western blot analysis. Full-length blots are presented in Figure S6. Quantification of (**J**,**K**,**I**) and (**M**,**N**,**L**) Error bars, SD (*n* = 3). (** *p* < 0.001, *p*-values between depicted groups).

To determine the role of p62 regulation in CAF activation, MRC5 cells were treated with p62-targeting small interfering RNAs (siRNAs) and exposed to hypoxia. Notably, p62 inhibition significantly attenuated both the mRNA and protein expression of *ACTA2*/αSMA, a hallmark of CAF activation (Figure 2A–C). The expression pattern of other CAF marker genes, such as fibroblast activation protein (FAP) and fibroblast-specific protein 1 (FSP1), also showed similar to that of *ACTA2* (Figure S2A,B). The mRNA and protein levels of αSMA remained suppressed to minimal levels in *p62^−/−^* mouse embryonic fibroblasts (MEFs), when exposed to hypoxia (Figure 2D,E). Moreover, p62-depleted MRC5 cells co-cultured with A549 cells failed to induce αSMA expression (Figure 2F), which suggested that p62 was essential for CAF activation. Next, to test whether autophagic degradation of p62 was required for CAF activation, we monitored autophagic flux by labeling LC3B puncta in CAFs co-cultured with A549 cancer cells. Co-culturing significantly increased the autophagic flux, and p62 inhibition effectively blocked the increase (Figure 2G,H). Next, we added 3-methyladenine (3MA), which blocks the early steps of the autophagic process by targeting type III phosphatidylinositol 3-kinases [27]. We found that 3MA counteracted both hypoxia-induced αSMA up-regulation and p62 loss (Figure 2I–K). Furthermore, we found that CAF activation was inhibited by the lysosome inhibitor, hydroxychloroquine (HCQ) [28], but not by the proteasome inhibitor, MG132 [29] (Figure 2L–N and Figure S2C). These findings suggested that p62 mediated CAF activation by enhancing the autophagic flux.

### 2.4. p62-Induced CAF Activation Promotes Early Tumorigenesis

**Figure 3:**
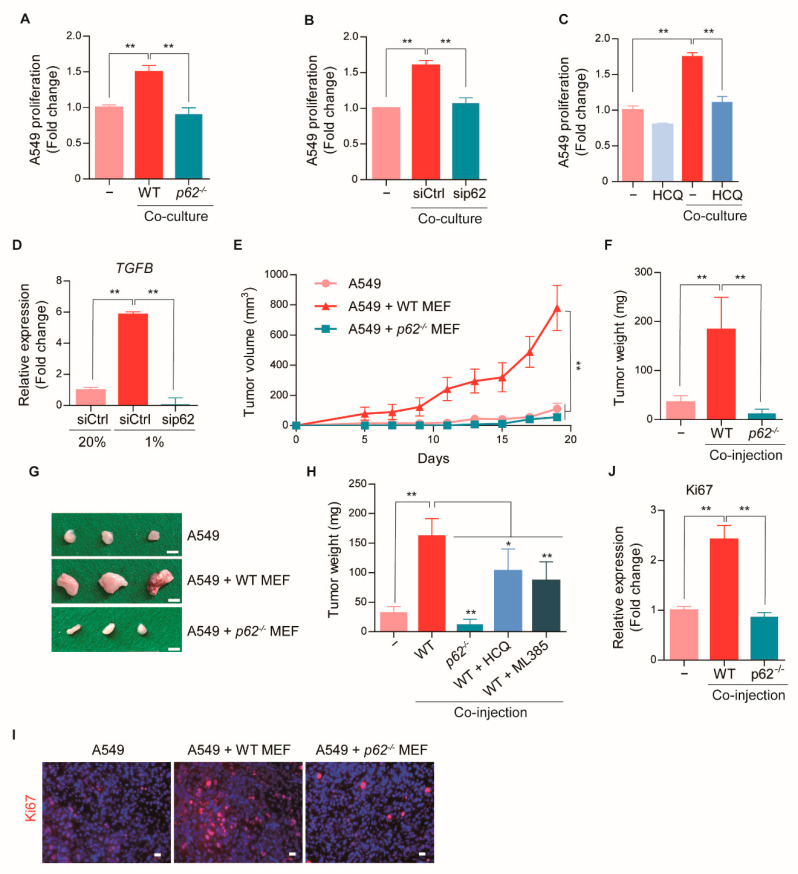
Inhibition of early tumorigenesis by deletion of p62 in CAFs. (**A**,**B**) The proliferation of A549-GFP cells was analyzed by measuring GFP expression after the co-culture with WT, (**A**) *p62^−/−^* MEFs, or (**B**) p62 knock-down MRC5 cells. Error bars, SD (*n* = 3). (**C**) The effect of HCQ (25 μM) on the proliferation of A549-GFP cells was analyzed by measuring GFP expression after the co-culture with MRC5 cells for 4 days. Error bars, SD (*n* = 3). (**D**) The mRNA expressions of *TGFB* after hypoxia (1% O_2_, 24 h) in WT or p62 knock-down MRC5 cells were analyzed by Real-time RT PCR. *GAPDH* was used as a reference gene in the analysis. Error bars, SD (*n* = 3). (**E**–**H**) The impact of hypoxia-exposed WT or *p62^−/−^* MEFs on tumor growth in A549 xenograft mouse models. A549 cells were injected alone or co-injected with 24 h hypoxia-exposed WT or *p62^−/−^* MEFs subcutaneously into nude mice (10 mice/each group). (**E**) Tumor volume was measured and calculated by use of the modified ellipsoid formula V = 1/2(Length × Width^2^). Error bars, SD (*n* = 10). (**F**) At the time of sacrifice, tumors were removed and weighted. Error bars, SD (*n* = 10). (**G**) Representative image of xenograft tumors. (**H**) After cell injection, HCQ (5 mg/kg, i.p.) or ML385 (5 mg/kg, i.p.) was administered daily Monday to Friday. At the time of sacrifice, tumors were removed and weighted. Error bars, SD (*n* = 10). (**I**) Ki67 immunofluorescence staining of xenograft tumor sections. The nuclei were stained with Hochest (blue). Scale bars: 20 μm. (**J**) Quantification of **I**. Error bars, SD (*n* = 3). (* *p* < 0.05, ** *p* < 0.001, *p*-values between depicted groups.).

Given our finding that p62 was transcriptionally induced and rapidly degraded via autophagy in CAFs derived from early stage cancers (Figure 1 and Figure 2), we investigated the role of p62-induced CAF activation in early tumor growth. Co-culture assays showed that the proliferation of GFP-labeled A549 cancer cells was significantly stimulated by wild-type MEFs, but not *p62^−/−^* MEFs (Figure 3A and Figure S2D). The essential role of p62 in CAF activation was confirmed in co-culture assays with p62-knockdown MRC5 cells (Figure 3B and Figure S2E). These data suggested that fibroblasts were differentiated into CAFs in response to cancer cells, and that p62 was an essential modulator. The importance of p62-dependent macroautophagy in CAF activation was independently confirmed by the finding that the autophagy inhibitor, HCQ, also efficiently inhibited CAF induction of cancer cell proliferation (Figure 3C). We next investigated the pathways involved in p62-mediated CAF activation. We found that *TGFB* was transcriptionally induced during CAF activation via p62-dependent macroautophagy (Figure 3D and Figure S2D). These results suggested that p62-induced CAF differentiation promoted cancer cell proliferation via TGFβ production.

To determine the role of p62 in CAF-mediated tumor growth, we generated a tumor xenograft mouse model. Immune-deficient nude mice were co-injected subcutaneously with A549 cancer cells and hypoxia-exposed wild-type or *p62^−/−^* MEFs. We used MEFs, which are proliferative and amenable for CAF activation, for in vivo study. Then, the tumor volumes were monitored every 3 days for 18 days to observe early tumorigenesis. The growth of A549 cancer cell tumors was drastically accelerated when co-injected with hypoxia-exposed MEFs (Figure 3E–H and Figure S3). At 18 days post injection, A549 cells co-injected with hypoxia-exposed CAFs generated tumors of greater volume (Figure 3E) and weight (Figure 3F), compared to tumors generated with injections of A549 cells alone. In sharp contrast, the efficacy of A549 cells to form tumors was almost completely abolished when co-injected with hypoxia-exposed *p62^−/−^* MEFs or treated with HCQ (Figure 3E–H and Figure S3). Moreover, the proliferation marker ki67 was contained far more in the tumors that developed in the presence of CAFs, when compared to tumors developing in the absence of CAFs (Figure 3I,J). However, in the presence of hypoxia-exposed *p62^−/−^* MEFs, the accumulations of ki67 in tumors were diminished. These results demonstrated that p62-mediated CAF activation promoted early tumorigenesis in lung adenocarcinoma, which implicated p62-dependent macroautophagy as a potential therapeutic target.

### 2.5. p62 Stimulates Nrf2 Signaling during CAF Differentiation

**Figure 4:**
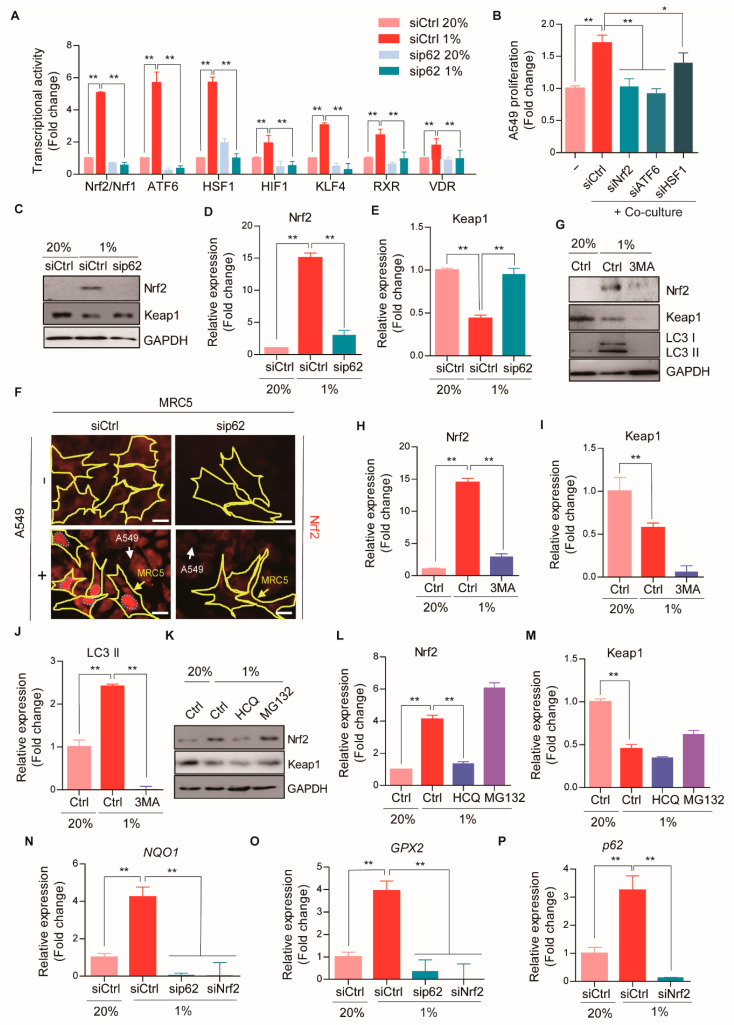
The p62-Nrf2 pathway activation during CAF differentiation. (**A**) The transcriptional activity of 45 pathways was measured using Cignal Finder 45-pathway Reporter Arrays after hypoxia (1% O_2_, 24 h) in WT or p62 knock-down MRC5 cells. Error bars, SD (*n* = 3). (**B**) The proliferation of A549-GFP cells was analyzed by measuring GFP expression after the co-culture with WT or Nrf2, ATF6, or HSF1 knock-down MRC5 cells. Error bars, SD (*n* = 3). (**C**) The protein expressions of Nrf2 after hypoxia (1% O_2_, 24 h) in WT or p62 knock-down MRC5 cells were assessed with Western blot analysis. Full-length blots are presented in Figure S6. (**D**,**E**) Quantification of (**C**). Error bars, SD (*n* = 3). (**F**) Representative images of immunofluorescence staining revealing Nrf2 expressions (red) in WT or p62 knock-down MRC5 cells (surrounded with yellow line) after the co-culture with A549-GFP cells (indicated with white arrows) for 4 days. Scale bars: 20 μm. (**G**–**M**) The protein expressions of Nrf2 after the treatment of (**G**) 3MA (5 mM, 24 h), (**K**) HCQ (25 μM, 24 h), or MG132 (5 μM, 24 h) were analyzed in hypoxic (1% O_2_, 24 h) MRC5 cells by Western blot analysis. Full-length blots are presented in Figure S6. Quantification of (**H**–**J**,**G**) and (**L**,**M**,**K**) Error bars, SD (*n* = 3). (**N**–**P**) The mRNA expressions of (**N**) *NQO1,* (**O**) *GPX2,* and (**P**) *P62* after hypoxia (1% O_2_, 24 h) in WT or p62 or Nrf2 knock-down MRC5 cells were analyzed by Real-time RT PCR. *GAPDH* was used as a reference gene in the analysis. Error bars, SD (*n* = 3). (* *p* < 0.05, ** *p* < 0.001, *p*-values between depicted groups).

To identify the pathway underlying p62-mediated CAF differentiation, we performed pathway analysis with the Cignal Finder 45-Pathway Reporter Array. We found potent transcriptional enhancement of at least seven signaling pathways, including Nrf2/Nrf1, ATF6, HSF1, HIF1α, KLF4, RXR, and VDR, in MRC5 fibroblasts during hypoxia-mediated CAF activation (Figure 4A). Strikingly, this transcriptional activation was abolished in p62-knockdown MRC5 cells (Figure 4A). Therefore, we further characterized the roles of these transcription factors in p62-mediated CAF activation. When A549-GFP cells were co-cultured with MRC5 cells, silencing Nrf2 almost completely blocked CAF-induced promotion of A549 proliferation (Figure 4B). A similar result was obtained when ATF6α was silenced (Figure 4B). These results suggested that p62 mediated CAF activation via a set of transcription factors whose transcriptional activity was selectively activated when fibroblasts were exposed to hypoxia and oxidative stress in the TME.

Because Nrf2 is a known downstream target of p62, we characterized the role of the Nrf2-p62 autophagy pathway in CAF activation. Consistent with the transcriptional activation of Nrf2 (Figure 4A), the Nrf2 protein level remarkably increased in hypoxia-induced CAFs, compared to normal fibroblasts (Figure 4C,D). The induction of Nrf2 was associated with the autophagic degradation of the E3 ligase, Keap1, by p62 (Figure 4C,E). The induction of Nrf2 and the degradation of Keap1 were abolished when p62 was depleted in hypoxia-exposed MRC5 cells (Figure 4C,E). Consistently, immunofluorescence assays showed that Nrf2 proteins accumulated in the nucleus of MRC5 CAFs co-cultured with cancer cells, in a manner strictly dependent on p62 (Figure 4F). Moreover, the accumulation of Nrf2 in hypoxia-induced CAFs was efficiently inhibited by the autophagy inhibitors, 3MA and HCQ, but not by the proteasome inhibitor, MG132 (Figure 4G–M). These results suggested that p62 mediated the autophagic degradation of Keap1, which led to Nrf2 induction, which in turn, facilitated the activation of normal fibroblasts to become CAFs.

We further characterized the role of Nrf2 in the metabolic reprogramming of CAFs by analyzing the expression of Nrf2 target genes, such as NAD(P)H quinone dehydrogenase 1 (*NQO1*) and glutathione peroxidase 2 (*GPX2*) [30,31]. Indeed, the transcription of *NQO1* and *GPX2* dramatically increased in hypoxia-mediated CAFs; conversely, these increases in transcription were not observed when p62 or Nrf2 was depleted (Figure 4N,O). Of note, the mRNA level of *p62* was markedly elevated in hypoxia-mediated CAFs, but not when Nrf2 was silenced (Figure 4P). Given that the *p62* promoter carries the antioxidant responsive element (ARE) sequence [32], this finding suggested that p62 and Nrf2 might form a positive feedback loop during CAF activation. Taken together, these results suggested that the p62-Nrf2 antioxidant pathway reacted to hypoxia- or cancer cell-mediated oxidative stresses by activating the cellular differentiation into the CAF phenotype, as a stress defense mechanism.

### 2.6. The p62-Nrf2 Pathway Is Required for CAF Activation

**Figure 5:**
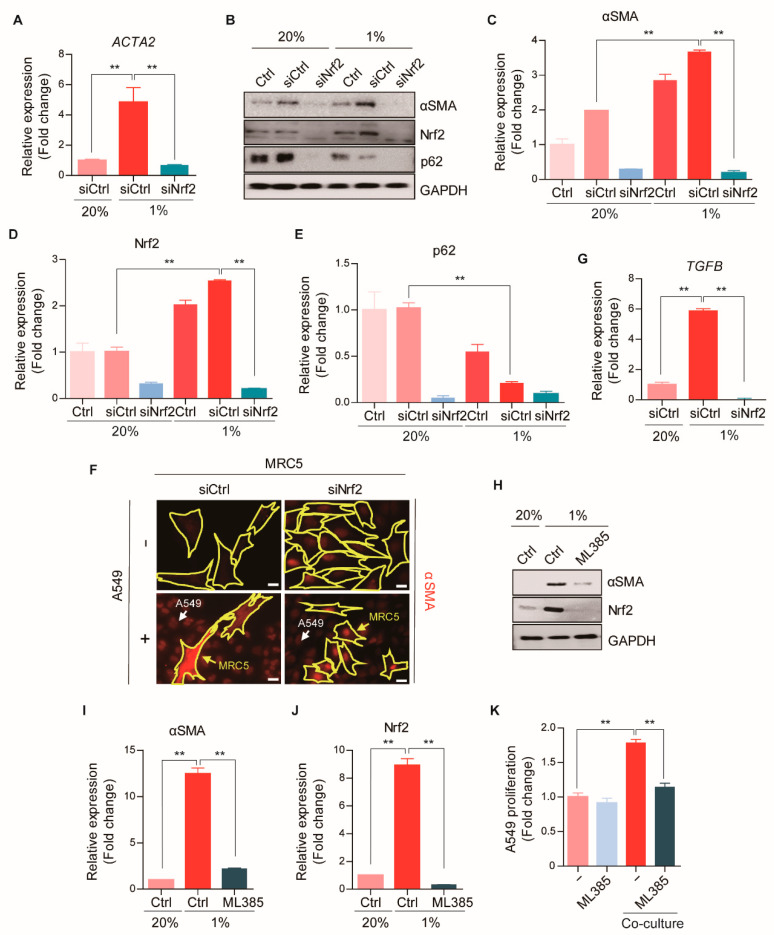
Inhibition of CAF activation by deletion of Nrf2. (**A**) The mRNA expressions of *ACTA2* after hypoxia (1% O_2_, 24 h) in WT or Nrf2 knock-down MRC5 cells were analyzed by Real-time RT PCR. *GAPDH* was used as a reference gene in the analysis. Error bars, SD (*n* = 3). (**B**) The protein expressions of αSMA after hypoxia (1% O_2_, 24 h) in WT or Nrf2 knock-down MRC5 cells were assessed with Western blot analysis. Full-length blots are presented in Figure S6. (**C**–**E**) Quantification of **B**. Error bars, SD (*n* = 3). (**F**) Representative images of immunofluorescence staining revealing αSMA expressions (red) in WT or Nrf2 knock-down MRC5 cells (surrounded with yellow line) after the co-culture with A549-GFP cells (indicated with white arrows) for 4 days. Scale bars: 20 μm. (**G**) The mRNA expressions of *TGFB* after hypoxia (1% O_2_, 24 h) in WT or Nrf2 knock-down MRC5 cells were analyzed by Real-time RT PCR. *GAPDH* was used as a reference gene in the analysis. Error bars, SD (*n* = 3). (**H**) The protein expressions of αSMA after the treatment of ML385 (5 μM, 24 h) in hypoxic (1% O_2_, 24 h) MRC5 cells were analyzed by Western blot analysis. Full-length blots are presented in Figure S6. (**I**,**J**) Quantification of **H**. Error bars, SD (*n* = 3). (**K**) The effect of ML385 (5 μM) on the proliferation of A549-GFP cells was analyzed by measuring GFP expression after the co-culture with MRC5 cells for 4 days. Error bars, SD (*n* = 3). (** *p* < 0.001, *p*-values between depicted groups).

We next determined the role of Nrf2 in CAF differentiation under cellular stress conditions in MRC5 fibroblasts co-cultured with A549 lung cancer cells. The knockdown of Nrf2 effectively counteracted the induction of αSMA under hypoxia, at both the mRNA (*ACTA2*) and protein (αSMA) levels (Figure 5A–E). Immunostaining analysis revealed that, during the differentiation of MRC5 cells into CAFs, αSMA accumulation was significantly attenuated by silencing Nrf2 expression (Figure 5F). The increase in *TGFB* transcription during CAF activation was also significantly down-regulated by Nrf2 silencing (Figure 5G). These results suggested that CAF activation was mediated by p62-Nrf2 signaling, which promoted TGFβ secretion and led to tumorigenesis.

We then utilized ML385, a ligand that inhibits Nrf2, to address the role of Nrf2 in CAF activation. As shown in Figure 5H–J, ML385 treatment significantly reduced the expression of αSMA in MRC5 cells under hypoxia. The failure to induce αSMA was associated with an impairment in the proliferation of A549 cancer cells (Figure 5K). It is known that ML385 synergistically enhances the anti-tumor efficacy of platinum-based drugs, doxorubicin and taxol, in Nrf2 activated lung cancer [33]. Due to somatic loss-of-function mutations in Keap1, Nrf2 is typically activated in lung cancer, particularly in adenocarcinomas [34]. Accordingly, targeting Nrf2 might be an effective strategy for suppressing both cancer cells and CAFs in lung adenocarcinoma. These results revealed that the activation of the p62-Nrf2 autophagy pathway mediated CAF differentiation, which led to tumorigenesis.

### 2.7. ATF6α Controls CAF Activation through the p62-Nrf2 Axis

**Figure 6:**
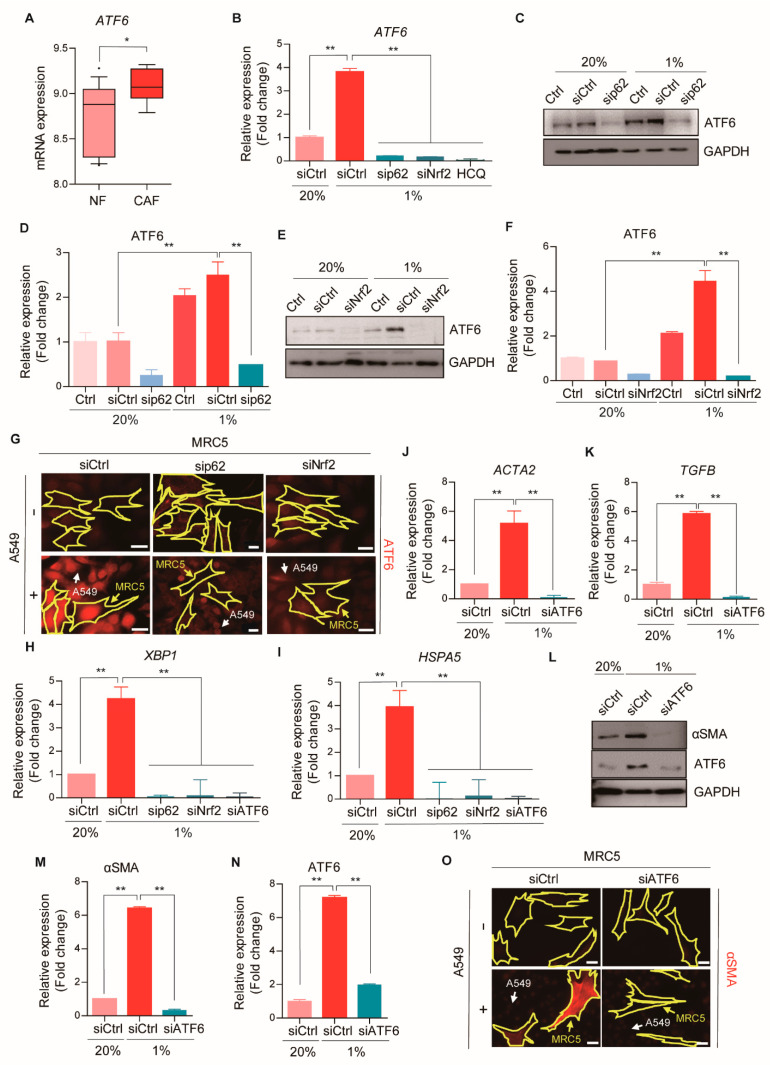
ATF6 α controls CAF activation through the p62-Nrf2 axis. (**A**) Analysis of *ATF6* mRNA expressions in the CAFs of 24 lung adenocarcinoma patients from [25]. Error bars, SD. (**B**) The mRNA expressions of *ATF6* after hypoxia (1% O_2_, 24 h) in WT, p62-, Nrf2-deficient, or HCQ-treated (25 μM, 24 h) MRC5 cells were analyzed by Real-time RT PCR. *GAPDH* was used as a reference gene in the analysis. Error bars, SD (*n* = 3). (**C**–**F**) The protein expressions of ATF6 after hypoxia (1% O_2_, 24 h) in WT or (**C**) p62 or (**E**) Nrf2 knock-down MRC5 cells were assessed with Western blot analysis. Full-length blots are presented in Figure S6. Quantification of (**D**,**C**) and (**F**,**E**). Error bars, SD (*n* = 3). (**G**) Representative images of immunofluorescence staining revealing ATF6 expressions (red) in WT or p62 or Nrf2 knock-down MRC5 cells (surrounded with yellow line) after the co-culture with A549-GFP cells (indicated with white arrows) for 4 days. Scale bars: 20 μm. (**H**,**I**) The mRNA expressions of (**H**) *XBP1* and (**I**) *HSPA5* after hypoxia (1% O_2_, 24 h) in WT or p62, Nrf2, or ATF6 knock-down MRC5 cells were analyzed by Real-time RT PCR. *GAPDH* was used as a reference gene in the analysis. Error bars, SD (*n* = 3). (**J**,**K**) The mRNA expressions of (**J**) *ACTA2* and (**K**) *TGFB* after hypoxia (1% O_2_, 24 h) in WT or ATF6 knock-down MRC5 cells were analyzed by Real-time RT PCR. *GAPDH* was used as a reference gene in the analysis. Error bars, SD (*n* = 3). (**L**) The protein expressions of αSMA after hypoxia (1% O_2_, 24 h) in WT or ATF6 knock-down MRC5 cells were assessed with Western blot analysis. Full-length blots are presented in Figure S6. (**M**,**N**) Quantification of **L**. Error bars, SD (*n* = 3). (**O**) Representative images of immunofluorescence staining revealing αSMA expressions (red) in WT or ATF6 knock-down MRC5 cells (surrounded with yellow line) after the co-culture with A549-GFP cells (indicated with white arrows) for 4 days. Scale bars: 20 μm. (* *p* < 0.05, ** *p* < 0.001, *p*-values between depicted groups.).

Our results showed that the transcriptional activity of ATF6 was potently enhanced in MRC5 fibroblasts during hypoxia-mediated CAF activation in a manner that depended on p62 (Figure 4A). Consistently, an examination of a public mRNA database [25] showed that the levels of *ATF6* mRNA were elevated in CAFs from patients with lung adenocarcinomas, compared to normal fibroblasts (Figure 6A). Therefore, we characterized the role of ATF6 in CAF activation. Indeed, *ATF6* mRNA expression was robustly induced in CAFs differentiated from hypoxia-exposed MRC5 fibroblasts (Figure 6B). This induction of *ATF6* under hypoxia was nearly completely blocked by silencing either p62 or Nrf2 (Figure 6B). The ATF6 protein level (Figure 6C–F) and its nuclear accumulation (Figure 6G) were also increased during CAF activation, and both were impaired with p62 or Nrf2 silencing. This induction of ATF6 strictly required autophagic flux, evidenced by its sensitivity to HCQ (Figure 6B). These results suggested that both the mRNA and protein levels of ATF6 were up-regulated during CAF differentiation through the p62-Nrf2 autophagic pathway.

Because ATF6 modulates the transcription of genes involved in the ER stress response [35], we analyzed signaling pathways related to ER stress during CAF activation. Indeed, the transcription of the X-box-binding protein 1 (*XBP1*) and *HSPA5* were robustly induced in hypoxia-mediated CAFs (Figure 6H,I). These inductions were not observed when p62 or Nrf2 was silenced (Figure 6H,I). These data suggested that, during CAF differentiation, hypoxia-associated oxidative stress triggered the ER stress response to promote protein folding and cell survival [36].

To investigate further whether ATF6 activation was required for CAF differentiation, we analyzed CAF activation in ATF6-silenced MRC5 fibroblasts. Indeed, silencing ATF6 attenuated the mRNA expression of *ACTA2* and *TGFB* under hypoxia, which indicated that CAF activation was blocked (Figure 6J,K). Consistently, silencing ATF6 also down-regulated αSMA protein expression during CAF activation (Figure 6L–O. In conclusion, ATF6 acted as a downstream target in the p62-Nrf2 pathway and promoted CAF differentiation by activating the ER stress response.

## 3. Discussion

To enhance tumor progression, cancer cells must recruit and reprogram non-malignant stromal cells to provide a tumor-supportive environment. We found that selective autophagy through p62 was a key factor in the conversion of normal fibroblasts into CAFs. Notably, the central signaling pathway in the TME, TGFβ, was empowered by the p62-autophagy axis. Our results expanded the well-known concept, that tumor growth requires CAF activation, by showing that a highly proliferative phenotype was achieved through selective autophagic degradation regulated by p62.

Our results suggested that p62 accumulated in malignant cells, but within CAFs, p62 was transcriptionally induced and then degraded by autophagy. Intriguingly, we observed a reciprocal relationship between the level of p62 protein in CAFs and the level in nearby cancer cells. This observation suggested that some type of communication between these two cell types might regulate the expression and degradation of p62. Given our results, one might speculate that p62 acts as a core mediator, which might reprogram CAFs to promote the secretion of key molecules that are required for communication with nearby malignant cells. Our findings showed that, during CAF activation, p62 regulated the activities of various transcription factors, including Nrf2/Nrf1, ATF6, HSF1, HIF1α, KLF4, RXR, and VDR, which involved dramatic alterations in their protein levels. These alterations affected the properties of CAFs and the proliferation of cancer cells (Figure 4A,B and Figure S4). Interestingly, HSF1, HIF1α, RXR, and VDR have been described as CAF modulators in various cancer types [4,17,37]. Here, we focused on the activity of Nrf2 and ATF6, because their transcriptional activities increased more than 5-fold during CAF activation. In future, the molecular mechanisms that underlie other transcription factors should be further elucidated, in both cancer cells and CAFs.

In contrast to recent findings, which suggested that p62 acted as a tumor suppressor in CAFs [16,17], this study showed that p62 acted as a tumor inducer that regulated autophagy and activated Nrf2. This discrepancy between studies might be explained by differences in the cancer types studied; we studied lung adenocarcinoma, which is known to display particularly high Nrf2 activation [34]. As the master regulator of anti-oxidant enzymes, Nrf2 is frequently altered in various types of cancer. Although Nrf2 activation is generally beneficial to health, due to its cytoprotective activity, persistent Nrf2 activation in cancer cells has deleterious effects on tumor suppressor activity, which leads to a poor prognosis in patients [38]. One could speculate that, because the lungs are highly exposed to free radicals, Nrf2 is particularly important in the airways and respiratory diseases. When Nrf2 is persistently and excessively activated, fibroblasts induce cellular senescence and the CAF phenotype, which leads to tumor growth [22]. In this context, when the TME is under intensive ROS-induced stress conditions, Nrf2 might be activated more proactively in lung fibroblasts than in other types of cells, via p62-dependent autophagy. This protective mechanism might give rise to their differentiation into CAFs.

Nrf2 regulation occurs at transcriptional and post-transcriptional levels. Post-transcriptional regulation includes Nrf2 protein stability and the availability of binding partners [39]. Our study showed that, during CAF activation, p62 modulated Nrf2 protein stability by enhancing autophagy and creating a positive feedback loop with its transcriptional targets. In addition, another positive feedback mechanism is present in the *Nrf2* promoter, which contains ARE-like sequences; these sequences are recognized by the Nrf2 protein, which then promotes its own transcription [39]. It remains to be determined how these multiple mechanisms are associated, but our data showed that *Nrf2* transcription was negatively regulated during CAF activation (Figures S1C and S5A,B). This finding suggested that the complementary activities of these mechanisms might result in the subsequent recovery of *p62* expression in the late stages of lung adenocarcinoma (Figure 1K).

Our work established ATF6 as a key signaling molecule, whose expression was regulated by the Nrf2-p62 pathway during CAF activation. The initial events during ER stress involves the activation of ATF6, which induces *XBP1* and *HSPA5* expression, which in turn, facilitate the unfolded protein response and autophagy [40,41,42]. During autophagic processes under stress conditions, overexpressed HSPA5 is arginylated on the N-terminus by arginyl transferase 1. The resulting arginylated HSPA5 binds to the ZZ domain of p62, which leads to the self-oligomerization of p62. This oligomerized p62, in complex with its cargoes, become targets to autophagosomes for selective lysosomal degradation [14,43]. In addition, under oxidative stress conditions, the N-terminal cysteines exposed on proteins become oxidized and arginylated, and these species are known to induce protein degradation [44]. Single N-terminal amino acids of a protein that promote the protein’s degradation are called N-degrons. The associated proteolytic system is called the N-degron pathway. Therefore, we speculate that the arginylated degrons exposed on HSPA5 or other proteins might activate p62 and link p62 to Keap1, and other cargoes, for lysosomal degradation. Further studies are needed to address these possibilities.

Our study showed that ATF6 was transcriptionally regulated by the Nrf2-p62 pathway during CAF activation. When the seven transcription factors identified in Figure 4A, were examined more precisely with real-time PCR, only *ATF6* and *HIF1A* were significantly transcriptionally modulated during hypoxia-induced CAF activation (Figure S5A,B). Interestingly, *HIF1A*, a key transcriptional regulator of the adaptive response to hypoxia, was also similarly regulated by the Nrf2-p62 pathway (Figure S5C). This process might be initiated by hypoxia-induced ROS generation during CAF activation, because Nrf2 is known to target a functional ARE in the *HIF1A* locus under oxidative stress conditions [45]. Thus, our results supported previous studies that showed that HIF1α played an important role in CAF activation [4,10]. Moreover, this finding provided a new perspective on how HIFα is regulated in CAFs.

From the therapeutic perspective, targeting CAFs in cancer therapy might provide advantages in treating primary tumors, recurrent tumors, and chemoresistance. In our mouse model, we showed that treatment with HCQ and ML385 could effectively block CAF activation and slow tumor progression (Figure 3C and Figure 5H–K, and Figure S3A–C). Thus, both autophagy and Nrf2 are attractive targets for treating cancer. HCQ is a lysosomal pH regulator. This drug was approved by the FDA for the treatment of malaria, rheumatoid arthritis, and several other diseases. Several studies have evaluated the ability of HCQ to enhance the efficacy of chemotherapy for treating lung cancer. HCQ treatment sensitized cancer cells to anti-cancer agents and enhanced the immune response; however, there is no definitive evidence that HCQ could be used as a single-agent anti-cancer drug [46]. Because HCQ has been used as a drug for decades, it might be readily available for a combination therapy in early phase clinical trials. In addition, we showed that the Nrf2 inhibitor, ML385, had the potential to slow progression, in both cancer cells and CAFs, particularly in Nrf2-activated lung adenocarcinoma.

In general, it is thought that CAF activation is irreversible. However, recent studies have indicated that the efficiency of anti-cancer drugs could be improved by reverting CAFs. CAF reversion might be achieved by targeting the pathways that underlie CAF activation. Indeed, CAF reprogramming has attracted much attention in colon and pancreatic cancers [47,48], where vitamin A and D were used to revert CAFs back to a quiescent state. In this context, our findings on the mechanisms underlying CAF activation might lead to the development of a novel anti-cancer therapy that reverts CAFs to a normal or quiescent phenotype.

**Figure 7:**
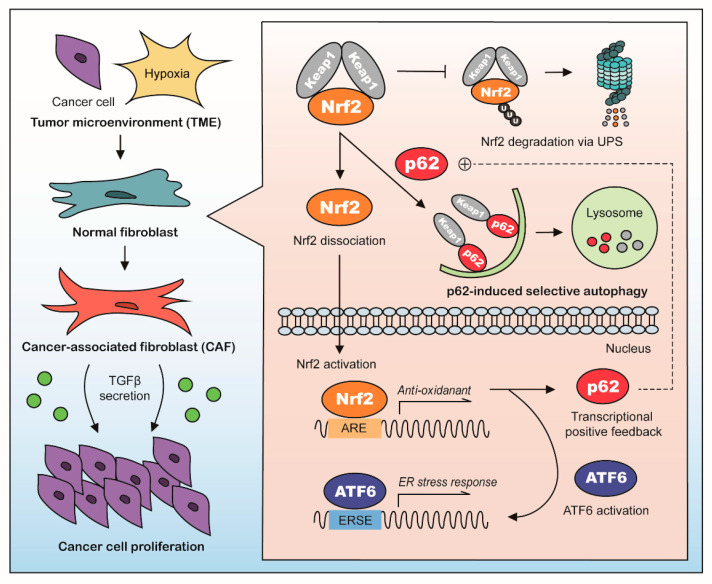
A schematic diagram showing how p62 mediates CAF activation.

In summary, this study demonstrated that CAF activation could be blocked by targeting the p62-autophagy-Nrf2-ATF6 axis (Figure 7). This finding could potentially lead to a selective preventive and/or therapeutic strategy for treating patients with lung adenocarcinoma.

## 4. Materials and Methods

### 4.1. Antibodies and Other Reagents

We used antibodies against the following proteins: GAPDH (Cat#sc-25778, Santa Cruz, Dallas, TX, USA), p62 (Cat#ab56416, Abcam, Cambridge, MA, USA), αSMA (Cat#ab5694, Abcam), Nrf2 (Cat#sc-365949, Santa Cruz), KEAP1 (Cat#sc-365626, Santa Cruz), ATF6 (Cat#sc-166659, Santa Cruz), and LC3B (Cat#ALX-803-081, Enzo Life Sciences, New York, NY, USA). Other reagents used in this study were 3-methyladenine (3MA, Cat#M9281, Sigma-Aldrich, St. Louis, MO, USA), hydroxychloroquine (HCQ, Cat#H0915, Sigma-Aldrich), MG132 (Cat#M8699, Sigma-Aldrich), and ML385 (Cat#SML1833, Sigma-Aldrich).

### 4.2. Tissue Microarray Analysis

Lung adenocarcinoma tissue microarrays were obtained from US Biomax (cat#LC1504, US Biomax, Derwood, MD, USA) for immunofluorescence. These 150 core tissue microarrays included samples from 50 cases of adenocarcinoma and matched cancer-adjacent normal lung tissues, with documentation on the clinical stages, pathological grades, and TNM classifications of the tumors. Paraffin-embedded tissues on the array slides were deparaffinized with two washes of xylene, with 3-min incubations for each wash. The sections were hydrated by incubating in successive ethanol mixtures of 100%, 95%, 70%, 50%, and 0% ethanol in distilled water (3 min each). Next, the slides were covered with antigen retrieval buffer (10 mM sodium citrate, 0.05% tween20, pH 6.0) and microwaved for 5 min, followed by cooling at room temperature for 20 min. Subsequently, the sections were rinsed in PBS twice, 5 min each, and they were blocked in 5% normal goat serum (NGS, Cat#5425, Cell Signaling Technology, Danvers, MA, USA) diluted in PBS and 0.3% Triton X-100 for 30 min. Next, the sections were stained with the primary antibodies diluted in 1% NGS in PBS solution at 4 °C overnight, followed by washing three times for 5 min in PBS. After the washing, the sections were incubated with Alexa Fluor-conjugated secondary antibodies (diluted in 1% NGS in PBS solution) for 1 h at room temperature. Nuclei were counterstained with the chromosomal dye, DAPI (Cat#D9542, Sigma Aldrich), and the arrays were mounted with a VectaMount Permanent Mounting Medium (Cat#H-5000, Vector Laboratories, Burlingame, CA, USA). Immunofluorescence was visualized with an inverted fluorescence microscope system (Zeiss AxioObserver, Zeiss, Jena, Germany), and images were analyzed with Zen 2.3 (Blue edition, Zeiss). The immunoreactivity score (IRS) of the samples was determined by multiplying the intensity of the staining and the percentage of stained area. Staining intensity was graded as follows: 0 (negative), 1 (weak), 2 (moderate), and 3 (strong). And the percentage scoring of immunoreactive tumor cells was as follows: 0 (0%), 1 (1–10%), 2 (11–50%), and 3 (>50%). p62 protein expression levels were divided into four groups based on the IRS values: negative (0), weak (1–4), moderate (6), and strong (9). αSMA expression levels were analyzed by classifying the IRS values: low (0–4) and high (6–9).

### 4.3. CAF Transcriptome Analysis

Data on the CAF gene expression profiles of 9 lung adenocarcinoma patients (6 for stage I/II and 3 for stage III) were obtained from the Gene Expression Omnibus (https://www.ncbi.nlm.nih.gov/geo/ (accessed on 20 February 2021), GSE22862 [25]). The gene expression levels in lung adenocarcinomas were compared to those from 15 normal lungs. The associations between the gene expressions and clinical stages were presented with a scatter plot or a box and whisker plot.

### 4.4. Cell Culture

Human fetal lung fibroblasts (MRC5, Cat#10171), human lung adenocarcinoma cells (A549, Cat#CCL-185), and GFP-labeled A549 cells (A549-GFP, Cat#MBS168483) were purchased from the Korean Cell Line Bank (Seoul, Korea), the American Type Culture Collection (Manassas, VA, USA), and MyBioSource (San Diego, CA, USA), respectively. *p62^+/+^* and *p62^−/−^* mouse embryonic fibroblasts (MEFs) were obtained from Keiji Tanaka’s laboratory (Tokyo Metropolitan Research Institute, Tokyo, Japan) with Tetsuro Ishii’s permission. Cells were cultured in DMEM (Cat#SH30243.01, Hyclone, Logan, UT, USA) supplemented with 10% FBS (Cat# SH30084.03, Hyclone) and 100 µg/mL penicillin/streptomycin. Cells were grown at 37 °C and 5% CO_2_. For knockout MEFs and stable cell lines, the absence or presence of the intended target protein(s) was confirmed with immunoblotting and/or immunocytochemistry.

### 4.5. CAF Activation and Cancer Cell Proliferation Assay

Normal fibroblasts, including MRC5 and MEF cells, were differentiated into CAFs by incubating in hypoxic conditions (1% O_2_) for 24 h, or co-culturing with cancer cells in normoxic conditions (20% O_2_). For co-culture experiments, normal fibroblasts were seeded with GFP-labeled A549 cancer cells at a 1:3 ratio (cancer cells: fibroblasts) and incubated for 4 days without refreshing the medium. CAF activation was confirmed by real-time PCR, immunoblotting, and/or immunocytochemistry of CAF markers (αSMA, FAP, or FSP1). In co-cultured cell experiments, the A549 cancer cells could be identified by detecting GFP fluorescence, and A549 cancer cell proliferation was evaluated by counting GFP-positive cells.

### 4.6. RNA Interference Analysis

Predesigned siRNAs (100 pmol) were transfected into MRC5 cells with Lipofectamine 3000 reagent (Cat#L3000015, Invitrogen, Waltham, MA, USA), according to the manufacturer’s instructions. We used the following siRNAs: siRNA negative control (Cat#sc-37007, Santa Cruz), sip62 (Cat#1144479, Bioneer, Daejeon, Korea), siNrf2 (Cat#sc-37030, Santa Cruz), siATF6 (Cat#sc-37699, Santa Cruz), siHSF1 (Cat#sc-35611, Santa Cruz), siHIF1 (Cat#sc-35561, Santa Cruz), and siRTN4 (Cat#sc-43974, Santa Cruz).

### 4.7. RNA Extraction and Quantitative Real-Time PCR

Total RNA was extracted from the cells with the ReliaPrep RNA Cell Miniprep system (Cat#Z6011, Promega, Madison, WI, USA), and cDNA was synthetized with TOPscript RT DryMIX (Cat#RT220, Enzynomics, Daejeon, Korea), according to the manufacturer’s instructions. Quantitative real-time PCR was performed with the iQ SYBR Green Supermix (Cat#1709990, Bio-Rad, Hercules, CA, USA) and the CFX-96 real-time PCR detection system (Bio-Rad). Primer pairs are listed in Table S1. CFX manager software (Bio-Rad) was used to calculate relative mRNA expression levels with the ΔΔCt method, normalized to the level of GAPDH mRNA expression in the same sample.

### 4.8. Western Blot Analysis

Cell lysates were prepared with the PRO-PREP Protein Extraction Solution (Cat#17081, iNtRON, Seongnam, Korea). Equivalent amounts of protein per sample (10 μg) were resolved on SDS-PAGE and transferred to polyvinylidene difluoride membranes. Blots were blocked with 5% skim milk in TBST solution (0.1% Tween-20 in Tris buffered saline). After washing with TBST twice, blots were incubated with primary antibodies (1:1000 dilution), then incubated with horseradish peroxidase-conjugated secondary antibodies (1:1000 dilution). Immunoreactive bands were detected with ECL reagents (Cat#34580, Thermo Fisher Scientific, Waltham, MA, USA). Densitometric analyses of Western blot bands were performed using Image J software (Image J 1.8, NIH, Bethesda, MD, USA).

### 4.9. Immunofluorescence Staining

To detect protein expression, cells were cultured on coverslips coated with poly-l-lysine. Cells were fixed with 4% paraformaldehyde in PBS (pH 7.4) for 15 min at room temperature, then washed three times with PBS for 5 min. After fixation, the cells were permeabilized with 0.5% Triton X-100 in PBS for 15 min. After washing three times with PBS, the cells were treated with blocking solution (2% bovine serum albumin [BSA] in PBS) for 1 h. Next, cells were incubated with primary antibodies, diluted in 2% BSA in PBS (1:50) overnight at 4 °C. Then, cells were washed three times for 10 min with PBS. Subsequently, the cells were incubated with Alexa Fluor-conjugated secondary antibody, diluted in 2% BSA in PBS, for 1 h at room temperature. VectaMount Permanent Mounting Medium (Cat#H-5000, Vector Laboratories), was used to mount the coverslips onto glass slides. Images were acquired with an inverted fluorescence microscope system (Zeiss AxioObserver, Zeiss) and analyzed with Zen 2.3 (Blue edition, Zeiss).

### 4.10. Dual Luciferase Reporter Gene Assay

Cignal Finder 45-Pathway Reporter Arrays (Cat#CCA-901 L, Qiagen, Hilden, Germany) were employed to identify the potential pathway regulated by p62 in CAF activation, according to the manufacturer’s instructions. Briefly, MRC5 cells were seeded on Cignal 45-pathway plates and transfected with p62 siRNA and 45-pathway luciferase reporter plasmids with Lipofectamine 3000 reagent (Cat#L3000015, Invitrogen). A complete media change was performed 24 h after transfection, and the cells were incubated in hypoxic conditions (1% O_2_) for another 24 h. Then, the luciferase activities of the cells were measured with a dual luciferase reporter assay kit (Cat#E1910, Promega). The reporter luciferase activity was normalized to renilla luciferase activity.

### 4.11. Tumor Xenograft Preparation and Histological Analysis

A549 cells (5 × 10^6^ cells), alone or mixed with 1 × 10^7^ hypoxic WT or *p62^−/−^* MEF cells (i.e., exposed to hypoxia for 24 h), were suspended in 100 μL PBS and subcutaneously injected into 6-week-old nude mice (BABL/c nude, DBL). Tumor tissues were measured once every three days with a caliper, and the tumor volume (mm^3^) was calculated according to the modified ellipsoid formula: V = 1/2(Length × Width^2^). After 18 days, the mice were sacrificed, and the tumors were weighed. For histology analysis, tumor tissues were fixed in formalin and embedded in paraffin and cut into 5 μm thick sections to be prepared for immunofluorescence staining.

### 4.12. Statistical Analysis

Data are presented as the mean ± standard deviation (SD) of three independent experiments. Significant differences were analyzed by one-way ANOVA and Turkey’s multiple comparisons test (GraphPad Prism 6, GraphPad Software, San Diego, CA, USA). For unpaired samples in CAF transcriptome analysis, two-tailed Mann-Whitney U test was used (GraphPad Prism 6). *p*-values < 0.05 were considered statistically significant.

## 5. Conclusions

p62-Nrf2 autophagy pathway plays a key role in the differentiation of normal fibroblasts into CAFs exposed to hypoxia and oxidative stresses under TME. Our results demonstrate that CAF activation absolutely requires targeted degradation of the E3 ligase Keap1 by p62 via macroautophagy. Degradation of Keap1 leads to the nuclear translocation of Nrf2 and transcriptional induction of antioxidant and adaptive responses. The transcriptional targets of Nrf2 during CAF activation include ATF6 and HIF1α, which mediates ER stress and hypoxia responses, respectively. Genetic or pharmacological inhibition of the Nrf2-ATF6 pathway abolishes CAF activation and tumor progression in mice. These results suggest that normal fibroblasts are differentiated into CAFs as protective responses to stresses under TME via the p62-Nrf2 pathway.

## Data Availability

Data sharing is not applicable to this article.

## Supplementary Materials

The following are available online at https://www.mdpi.com/2072-6694/13/4/864/s1, Figure S1: p62 expression patterns in human lung adenocarcinoma, Figure S2: p62 is required for CAF activation to promote cancer cell proliferation, Figure S3: Effect of blocking CAFs by targeting the p62-Nrf2 autophagy pathway on tumor growth in A549 xenograft mouse models, Figure S4: HIF1α and RXR inhibitions during CAF activation attenuate cancer cell proliferation, Figure S5: *ATF6* and *HIF1A* mRNA expressions are modulated through the p62-Nrf2 axis, Figure S6: Full-length Western blots, Table S1: Primers used for real-time PCR.
