# Supplementary material for: p62-Induced Cancer-Associated Fibroblast Activation via the Nrf2-ATF6 Pathway Promotes Lung Tumorigenesis
Source: Cancers (Basel). 2021 Feb 18;13(4):864. doi: 10.3390/cancers13040864 (PMC7922306; doi:10.3390/cancers13040864)
Supplement: Supplementary file 1 [file cancers-13-00864-s001.pdf]

cancers

Supplementary Materials: p62-Induced Cancer-Associated Fibroblast Activation via the Nrf2-ATF6 Pathway Promotes Lung Tumorigenesis

Ji In Kang, Dong Hyun Kim, Ki Woon Sung, Sang Mi Shim, Hyunjoo Cha-Molstad, Nak Kyun Soung, Kyung Ho Lee, Joonsung Hwang, Hee Gu Lee, Yong Tae Kwon and Bo Yeon Kim

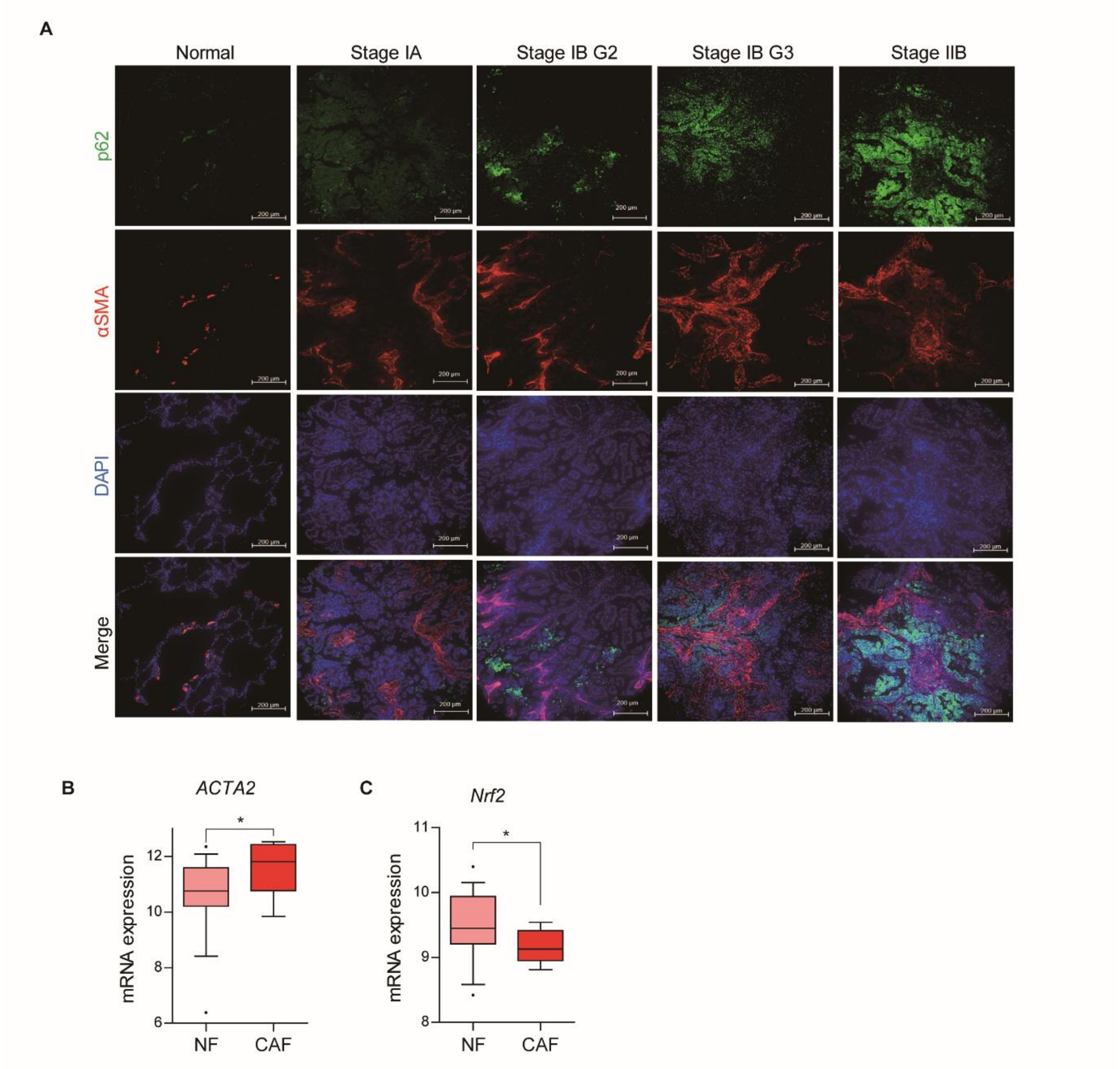

Figure S1. p62 expression patterns in human lung adenocarcinoma. (**A**) Immunofluorescence analysis of p62 expressions in the CAFs of 46 stage I/II lung adenocarcinoma patients using a tissue array. Representative images showing the expressions of p62 (green) and αSMA (red) by the cancer stages. The nuclei were stained with DAPI (blue). Scale bars: 200 µm. (**B**,**C**) Analysis of (**B**) *ACTA2* and (**C**) *NFE2L2* mRNA expressions in the CAFs compared to normal fibroblasts of 24 lung adenocarcinoma patients from [1]. Error bars, SD. (*$p < 0.05$, p-values between depicted groups.).

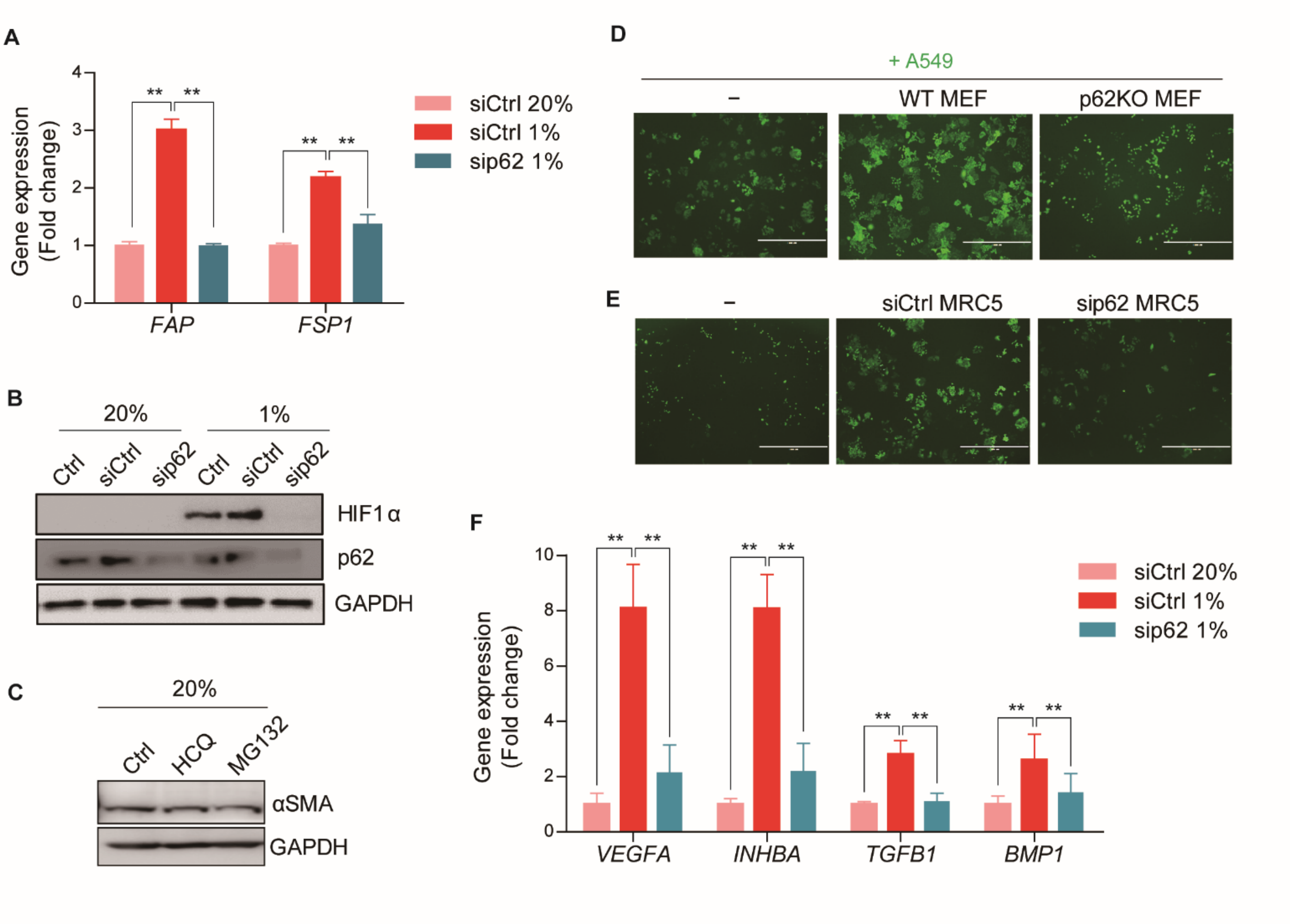

Figure S2. p62 is required for CAF activation to promote cancer cell proliferation. (**A**) The mRNA expressions of CAF markers *AFAP* and FSP1 after hypoxia (1% O_2, 24 h) in WT or p62 knock-down MRC5 cells were analyzed by Real-time RT PCR. *GAPDH* was used as a reference gene in the analysis. Error bars, SD ($n = 3$). (**B**) The protein expressions of HIF1α and p62 after hypoxia (1% O_2, 24 h) in WT or p62 knock-down MRC5 cells were assessed with Western blot analysis. The up-regulation of HIF1α after hypoxia and deletion of p62 expression in p62 knock-down cells were confirmed. (**C**) The protein expressions of αSMA after the treatment of HCQ (25 μM, 24 h) or MG132 (5 μM, 24 h) upon normoxic conditions (1% O_2, 24 h) were analyzed in MRC5 cells by Western blot analysis. The result showed no significant change. (**D**,**E**) The proliferation of A549-GFP cells was analyzed by measuring GFP expression after the co-culture with WT, (**D**) *p62*$^{-/-}$ MEFs, or (**E**) p62 knock-down MRC5 cells. Representative images showing the GFP expressions (green). (**F**) The cytokine gene expression profiling analysis were performed with the Human Cytokine RT 2 Profiler PCR Array (Qiagen, Hilden, Germany) after hypoxia (1% O_2, 24 h) in WT or p62 knock-down MRC5 cells. Error bars, SD ($n = 3$). (** $p < 0.001$, p-values between depicted groups.).

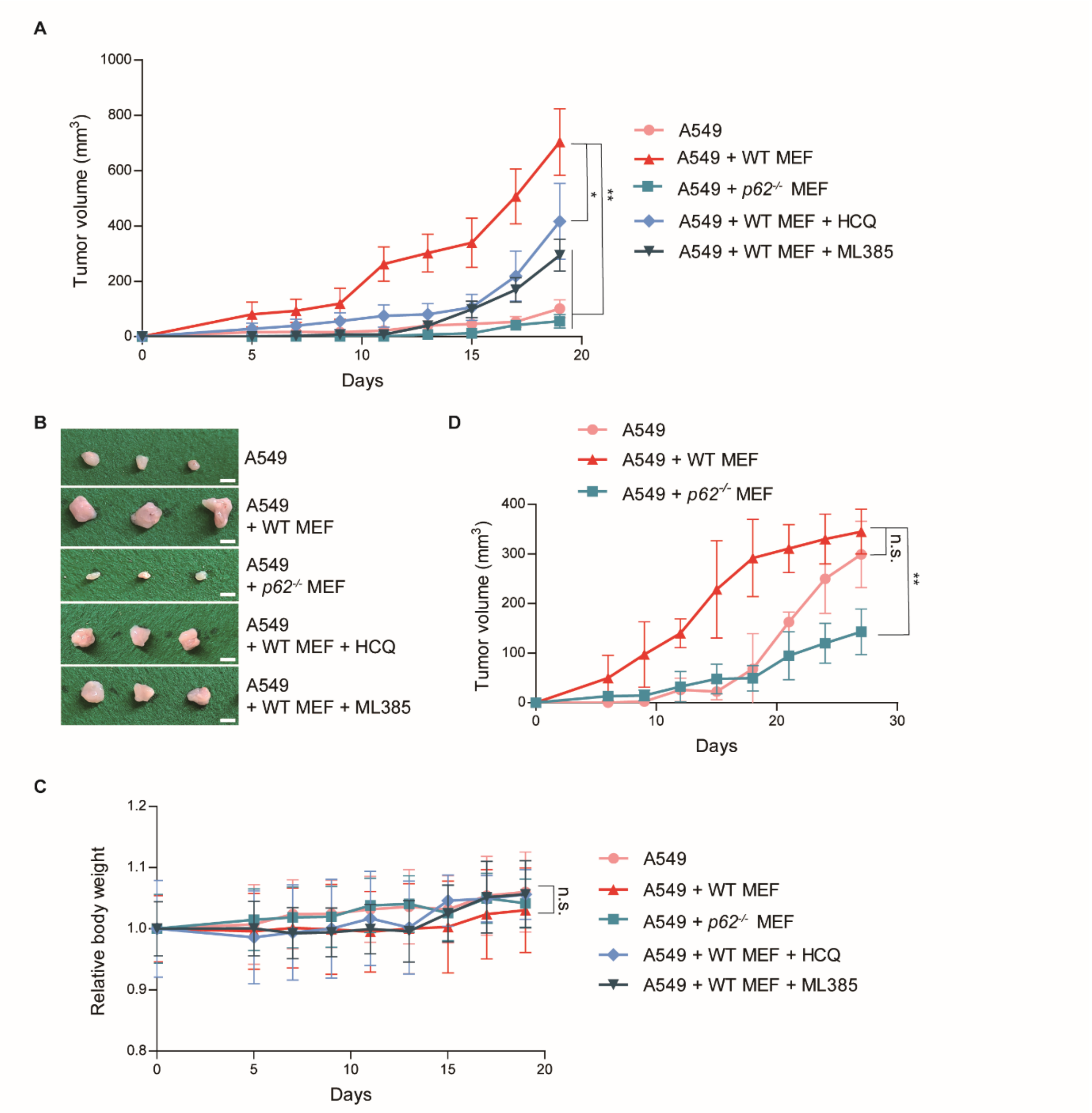

Figure S3. Effect of blocking CAFs by targeting the p62-Nrf2 autophagy pathway on tumor growth in A549 xenograft mouse models. (**A**–**D**) A549 cells were injected alone or co-injected with 24 h hypoxia-exposed WT or *p62*$^{-/-}$ MEFs subcutaneously into nude mice (10 mice/each group). Then, HCQ (5 mg/kg, i.p.) or ML385 (5 mg/kg, i.p.) was administered daily Monday to Friday. (**A**) Tumor volume was measured and calculated by use of the modified ellipsoid formula V = 1/2 (Length × Width^2). Error bars, SD (n = 10). (**B**) Representative image of xenograft tumors. (C) Changes in the mean of body weight over the time course of the experiment are shown. Error bars, SD (n = 10). (**D**) At the time of sacrifice, tumors were removed and weighted. At 27 days post injection, A549 tumor volumes became similar to the A549 + WT MEF tumor volumes, while A549 + *p62*$^{-/-}$ MEFs tumor were still small. Error bars, SD (n = 10). (* $p < 0.05$, ** $p < 0.001$, n.s. $p > 0.05$, *p*-values between depicted groups.).

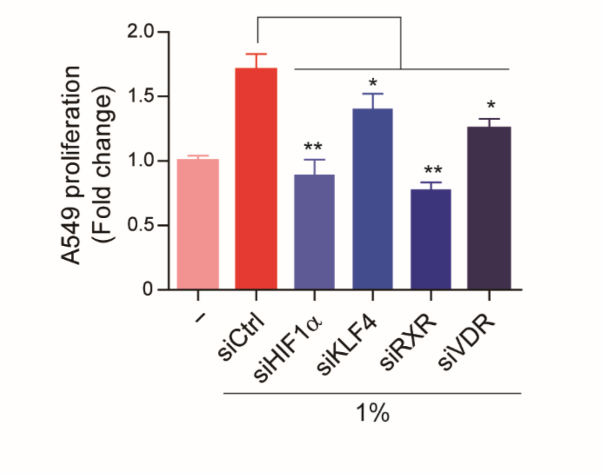

Figure S4. HIF1α and RXR inhibitions during CAF activation attenuate cancer cell proliferation. The proliferation of A549-GFP cells was analyzed by measuring GFP expression after the co-culture with WT or HIF1α, KLF4, RXR, or VDR knock-down MRC5 cells. Particularly, HIF1α and VDR inhibition significantly (** $p < 0.001$) suppressed the A549-GFP cell proliferation. Error bars, SD ($n = 3$). (* $p < 0.05$, ** $p < 0.001$, p-values between depicted groups.).

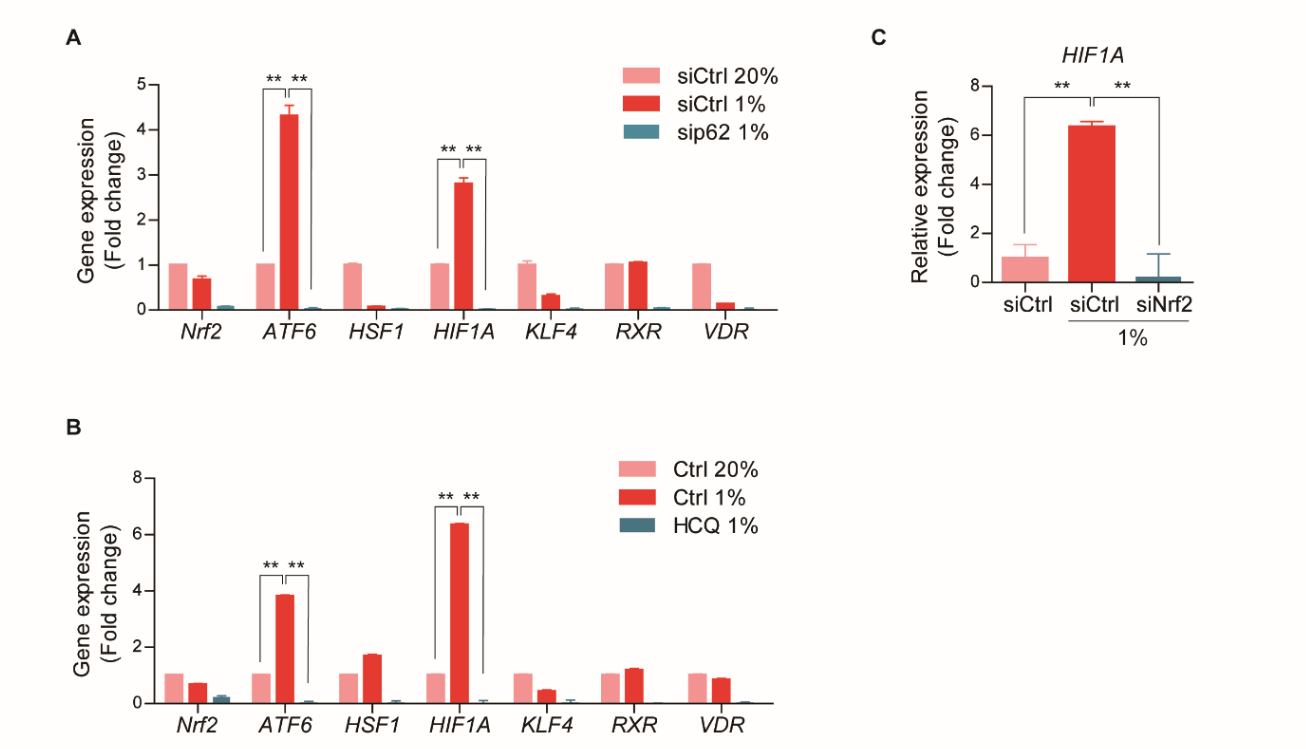

Figure S5. *ATF6* and *HIF1A* mRNA expressions are modulated through the p62-Nrf2 axis. (**A–C**) The mRNA expressions of indicated genes were analyzed by Real-time RT PCR, and *GAPDH* was used as a reference gene in the analysis. (**A**) The mRNA expressions were measured after hypoxia (1% O_2, 24 h) in WT or p62 knock-down MRC5 cells. Error bars, SD ($n = 3$). (**B**) The mRNA expressions were measured after hypoxia (1% O_2, 24 h) in MRC5 cells with or without HCQ treatment (25 μM, 24 h). Error bars, SD ($n = 3$). **(C)** The mRNA expressions of *HIF1A* were measured after hypoxia (1% O_2, 24 h) in WT or Nrf2 knock-down MRC5 cells. Error bars, SD ($n = 3$). (** $p < 0.001$, p-values between depicted groups.).

Fig.1M

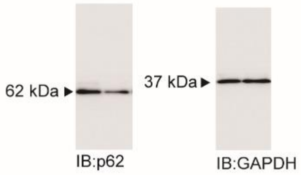

Fig.2B

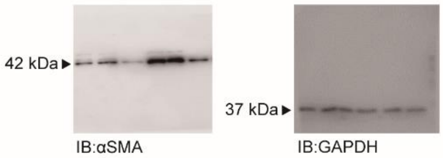

Fig.2D

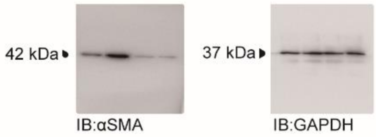

Fig.2I

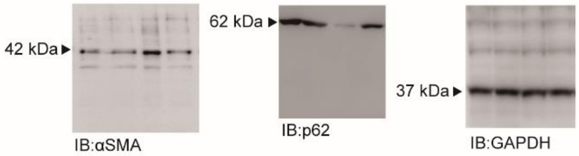

Fig.2L

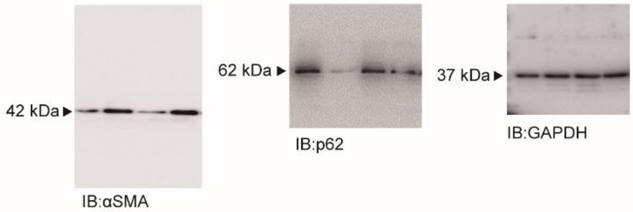

Fig.4C

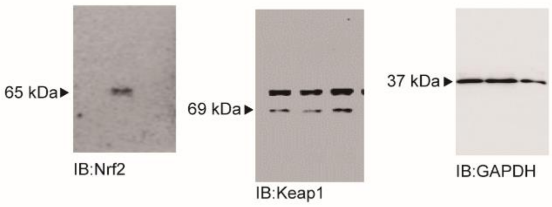

Fig.4G

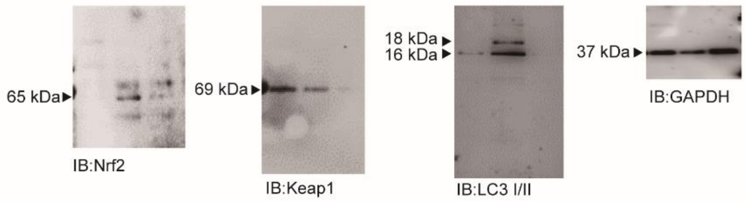

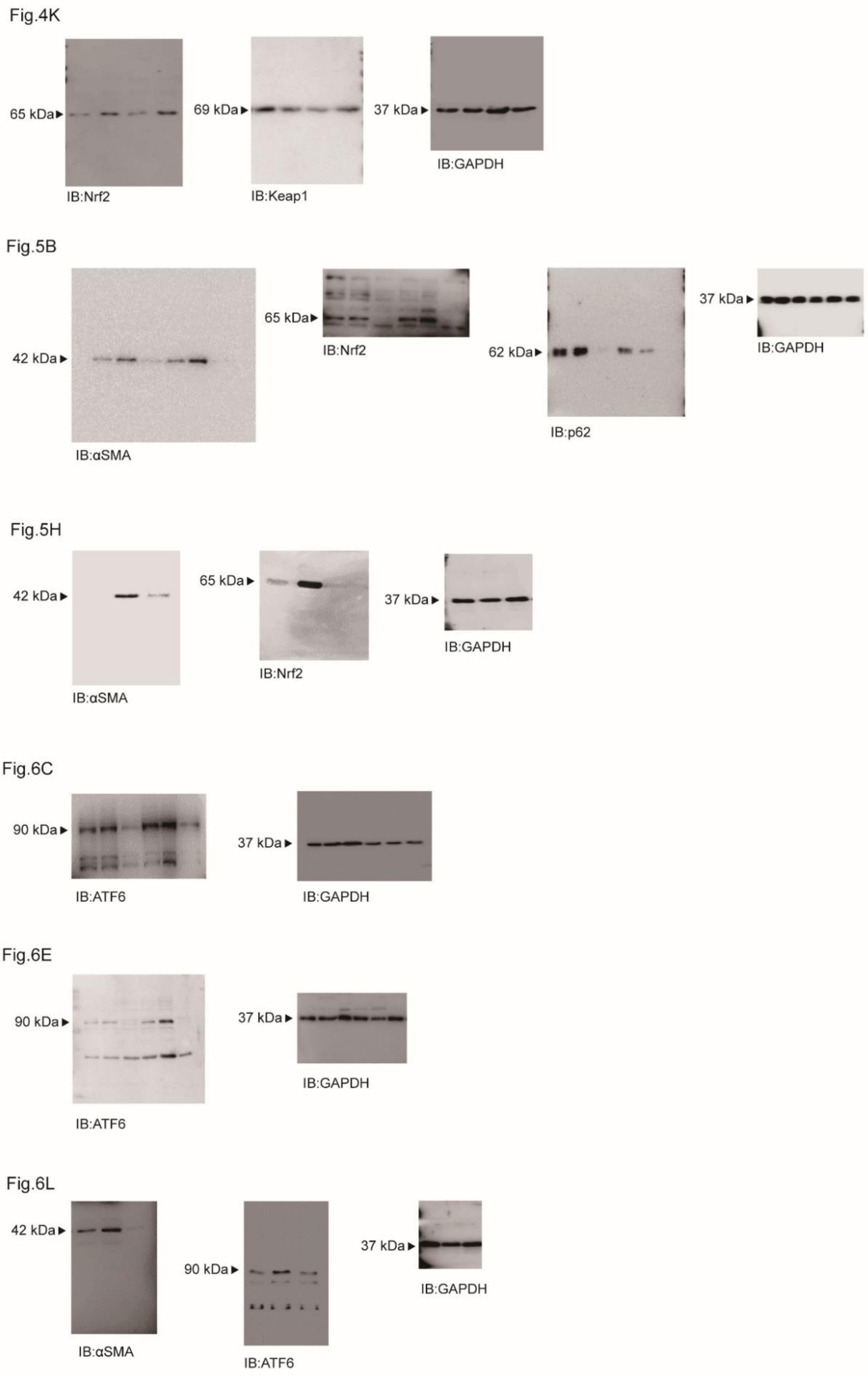

Figure S6. Full-length Western blots.

Table S1. Primers used for real-time RT PCR.

Target Gene	Primer Sequence (5′-3′)	
	**Forward**	**Reverse**
ATCA2	AAAAGACAGCTACGTGGGTGA	GCCATGTTCTATCGGGTACTTC
p62	GCACCCCAATGTGATCTGC	CGCTACACAAGTCGTAGTCTGG
TGFB	GGCCAGATCCTGTCCAAGC	GTGGGTTTCCACCATTAGCAC
NQO1	GAAGAGCACTGATCGTACTGGC	GGATACTGAAAGTTCGCAGGG
GPX2	GGTAGATTTCAATACGTTCCGGG	TGACAGTTCTCCTGATGTCCAAA
ATF6	TCCTCGGTCAGTGGACTCTTA	CTTGGGCTGAATTGAAGGTTTTG
XBP1	CCCTCCAGAACATCTCCCCAT	ACATGACTGGGTCCAAGTTGT
HSPA5	CATCACGCCGTCCTATGTCG	CGTCAAAGACCGTGTTCTCG
Nrf2	TCAGCGACGGAAAGAGTATGA	CCACTGGTTTCTGACTGGATGT
HSF1	CCATGAAGCATGAGAATGAGGC	CTTGTTGACGACTTTCTGTTGC
KLF4	CCCACATGAAGCGACTTCCC	CAGGTCCAGGAGATCGTTGAA
HIF1	GAACGTCGAAAAGAAAAGTCTCG	CCTTATCAAGATGCGAACTCACA
RXR	ATGGACACCAAACATTTCCTGC	GGGAGCTGATGACCGAGAAAG
VDR	GTGGACATCGGCATGATGAAG	GGTCGTAGGTCTTATGGTGGG
